# Aspirin in Primary Prevention: Looking for Those Who Enjoy It

**DOI:** 10.3390/jcm13144148

**Published:** 2024-07-16

**Authors:** Roberta Della Bona, Simona Giubilato, Marco Palmieri, Stefano Benenati, Roberta Rossini, Stefania Angela Di Fusco, Filippo Novarese, Giuseppe Mascia, Nicola Gasparetto, Antonio Di Monaco, Laura Gatto, Filippo Zilio, Carlotta Sorini Dini, Francesco Borrello, Giovanna Geraci, Carmine Riccio, Leonardo De Luca, Furio Colivicchi, Massimo Grimaldi, Michele Massimo Giulizia, Italo Porto, Fabrizio Giovanni Oliva

**Affiliations:** 1 Cardiovascular Disease Unit, IRCCS Ospedale Policlinico San Martino, 16132 Genoa, Italy; giuseppe_mascia@virgilio.it (G.M.); italo.porto@unige.it (I.P.); 2 Cardiology Department, Cannizzaro Hospital, 95126 Catania, Italy; 3 Department of Internal Medicine (Di.M.I.), University of Genova, 16132 Genoa, Italy; stefanobenenatimd@gmail.com (S.B.); filipponovarese1@gmail.com (F.N.); 4 Division of Cardiology, Emergency Department and Critical Areas, Azienda Ospedaliera Santa Croce e Carle, 12100 Cuneo, Italy; rossini.r@ospedale.cuneo.it; 5 Cardiology Department, San Filippo Neri Hospital, ASL Roma 1, 00135 Rome, Italy; stefaniaa.difusco@aslroma1.it (S.A.D.F.); furio.colivicchi@aslroma1.it (F.C.); 6 Division of Cardiology, AULSS2 Marca Trevigiana, Ca’ Foncello Hospital, 31100 Treviso, Italy; nicola.gasparetto@aulss2.veneto.it; 7 Department of Cardiology, General Regional Hospital “F. Miulli”, Acquaviva delle Fonti, 70021 Bari, Italy; a.dimonaco@gmail.com (A.D.M.); m.grimaldi@miulli.it (M.G.); 8 Cardiology Department, San Giovanni Addolorata Hospital, 00184 Rome, Italy; lauragatto81@gmail.com; 9 Department of Cardiology, Santa Chiara Hospital, APSS, 2, Largo Medaglie d’Oro, 38123 Trento, Italy; filippo.zilio@apss.tn.it; 10 Division of Cardiology, Department of Medical Biotechnologies, University of Siena, 53100 Siena, Italy; carlotta.sorinidini@ao-siena.toscana.it; 11 Division of Cardiology and Intensive Care Unit, Pugliese-Ciaccio Hospital, 88100 Catanzaro, Italy; fborrello@hotmail.com; 12 Cardiology Unit, S. Antonio Abate Hospital, ASP Trapani, 91016 Erice, Italy; giovannageraci@hotmail.com; 13 Cardiovascular Department, Sant’Anna e San Sebastiano Hospital, 81100 Caserta, Italy; carmine.riccio8@icloud.com; 14 Division of Cardiology—Fondazione IRCCS Policlinico San Matteo, 27100 Pavia, Italy; l.deluca@smatteo.pv.it; 15 Cardiology Department, Garibaldi Nesima Hospital, 95122 Catania, Italy; michele.gulizia60@gmail.com; 16 “A. De Gasperis” Cardiovascular Department, Division of Cardiology, ASST Grande Ospedale Metropolitano Niguarda, Piazza dell’Ospedale Maggiore 3, 20162 Milan, Italy; fabrizio.oliva@ospedaleniguarda.it

**Keywords:** aspirin, primary prevention, cardiovascular risk, platelet, bleeding, thrombosis, diabetes, guidelines, cancer

## Abstract

Based on a wealth of evidence, aspirin is one of the cornerstones of secondary prevention of cardiovascular disease. However, despite several studies showing efficacy also in primary prevention, an unopposed excess risk of bleeding leading to a very thin safety margin is evident in subjects without a clear acute cardiovascular event. Overall, the variability in recommendations from different scientific societies for aspirin use in primary prevention is a classic example of failure of simple risk stratification models based on competing risks (atherothrombosis vs. bleeding), perceived to be opposed but intertwined at the pathophysiological level. Notably, cardiovascular risk is dynamic in nature and cannot be accurately captured by scores, which do not always consider risk enhancers. Furthermore, the widespread use of other potent medications in primary prevention, such as lipid-lowering and anti-hypertensive drugs, might be reducing the benefit of aspirin in recent trials. Some authors, drawing from specific pathophysiological data, have suggested that specific subgroups might benefit more from aspirin. This includes patients with diabetes and those with obesity; sex-based differences are considered as well. Moreover, molecular analysis of platelet reactivity has been proposed. A beneficial effect of aspirin has also been demonstrated for the prevention of cancer, especially colorectal. This review explores evidence and controversies concerning the use of aspirin in primary prevention, considering new perspectives in order to provide a comprehensive individualized approach.

## 1. Introduction

Salicylin has been known for its analgesic properties for thousands of years, but it was only in 1897 that acetylsalicylic acid was synthesized as such, marketed as “aspirin”, and used for pain-relieving and antipyretic purposes. It took even longer to find out about the platelet inhibition properties of the compound. According to a review article from the Antiplatelet Trialists’ Collaboration dating back to 1988, in a pooled and heterogeneous cohort of patients with established cardiovascular disease (CVD), aspirin resulted in a 15% reduction in vascular mortality and a 30% reduction in non-fatal cardiovascular events [1]. Since then, aspirin has become the mainstay for the secondary prevention of cardiovascular events [2,3]. By contrast, whether a similar benefit could be expected in patients at high risk but without formal diagnosis of CVD has instead remained debated. Nonetheless, the use of aspirin for primary prevention of CVD is widespread, with more than one out of three adults aged ≥50 years and almost one out of two aged ≥70 years taking aspirin, without any previous cardiovascular event, as revealed by an interview of the U.S. population in 2017 [4]. This conflicts with the inconsistent results of seminal trials and guidelines recommendations.

This review provides an overview of the current evidence, recommendations and controversies concerning the use of aspirin in primary prevention and offers new perspectives to guide the best individualized approach.

## 2. Pharmacology of Aspirin: Joys and Sorrows

Aspirin diffuses through the gastric and duodenal mucosa, entering the portal circulation where platelets are exposed to it. There are two different formulations available for clinical use: immediate release, which reaches a plasma peak by 15 min and is particularly useful in the case of acute events, and gastro-resistant tablets, which reach a plasma peak by 3–4 h and are indicated for chronic treatment [5]. Despite the short half-life of aspirin (15–20 min), the effect on platelets is permanent. This is because platelets are anucleate cellular residues and the regeneration of target enzymes requires the production of new platelets [5,6]. Absorption may be influenced not only by the formulation and dosage, but also by the presence or absence of food and by gastric pH [5]. 

The therapeutic role of aspirin relies on the inhibition of the arachidonic acid cycle. Arachidonic acid is the substrate of cyclooxygenases 1 and 2 (COX-1 and COX-2) which transform it into the intermediate prostaglandin H2 (PGH2). PGH2 is then transformed by tissue-specific enzymes into several derivatives, including thromboxane and prostacyclin G2 (PGI2). Thromboxane promotes platelet aggregation and has a vasoconstrictor and proatherogenic effect [7]. In contrast, PGI2 inhibits platelet aggregation, induces vasodilation, and has a cytoprotective effect on the gastric mucosa [8,9]. Aspirin acts by acetylating a serine residue of COXs enzymes, thereby preventing arachidonic acid from accessing the catalytic site. Thromboxane comes mostly from the action of COX-1, the only isoform expressed on mature platelets, while PGI2 mainly derives from the action of COX-2 [10,11]. Irreversible inhibition of COX-1 takes place already at low doses of aspirin, so production of thromboxane is largely suppressed after a week of cumulative treatment [10,12,13]. In contrast, inhibition of COX-2 takes place at higher aspirin doses, so that PGI2-mediated vascular effects (such as blood pressure control and renin-angiotensin system inhibitor function) are not, or at most marginally, affected by low-dose aspirin [12,14,15]. Nonetheless, because the relationship between platelet thromboxane production and thromboxane-induced platelet aggregation is not linear and small concentrations of thromboxane can result in platelet aggregation, at least a 95% inhibition of COX-1 activity must be achieved to affect platelet activity [16]. In this regard, some authors have proposed the debated concept of aspirin resistance, which may be explained by the inter-individual variability in response to aspirin. In addition to clinical conditions later explored, this may be partially attributable to reduced bioavailability of aspirin [17], to genetic polymorphisms of COX-1, and to the residual protein synthesis activity of platelets [18], including COX-1 formation [19]. Given this background, aspirin treatment regimens generally require daily administration of 75–100 mg of the drug. 

In addition, aspirin also interferes with the production of inflammatory cytokines, growth factors, and other mediators [6,20,21]. In particular, the COX-2 pathway participates in several pathophysiological processes, including carcinogenesis. COX-2 is overexpressed in cancer precursors in the bowel, lung, breast, and many other organs [22], and COX-2 signalling is deeply involved in angiogenesis, stromal cell regulation, and inflammation [23]. This strongly supports a potential preventive role for aspirin in this context.

## 3. Deep Diving in Data: How Effective and Safe Is Aspirin?

**Figure 1:**
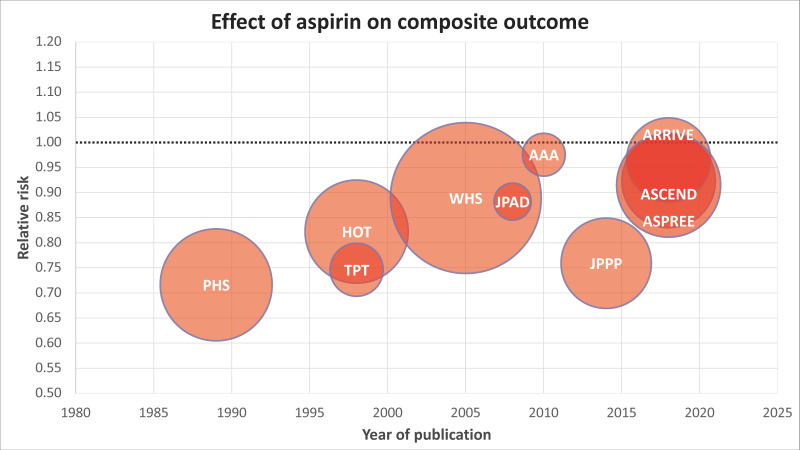
Timeline of randomized trials investigating aspirin use in primary prevention. Trials are presented as circles whose area is proportional to the sample size and are ordered on the x axis according to the year of publication. The main study result is graphically presented as the relative risk for a primary composite study endpoint, which is spread along the y axis, defined as a composite of fatal and nonfatal myocardial infarction plus fatal and nonfatal ischemic stroke. The dotted line intersecting the y axis indicates a relative risk of 1 (equivalence between treatments). Data from British Doctor Study (BDS), Primary Prevention Project (PPP), The Prevention Of Progression Of Arterial Disease And Diabetes (POPADAD), and International Polycap Study-3 (TIPS-3) are not reported in this analysis due to lack of data of separate outcome. Abbreviations. PHS: Physicians’ Health Study (relative risk, RR 0.72); HOT: Hypertension Optimal Treatment (RR 0.82); TPT: Thrombosis Prevention Trial (RR 0.75); WHS: Women’s Health Study (RR 0.89); JPAD: Low-dose aspirin for primary prevention of atherosclerotic events in patients with type 2 diabetes (RR 0.88); AAA: Aspirin for Asymptomatic Atherosclerosis (RR 0.98); JPPP: Japanese Primary Prevention Project (RR 0.76); ASCEND: A Study of Cardiovascular Events in Diabetes (RR 0.92); ARRIVE: Aspirin to Reduce Risk of Initial Vascular Events (RR 0.97); ASPREE: Aspirin in Reducing Events in the Elderly (RR 0.91).

**Figure 2:**
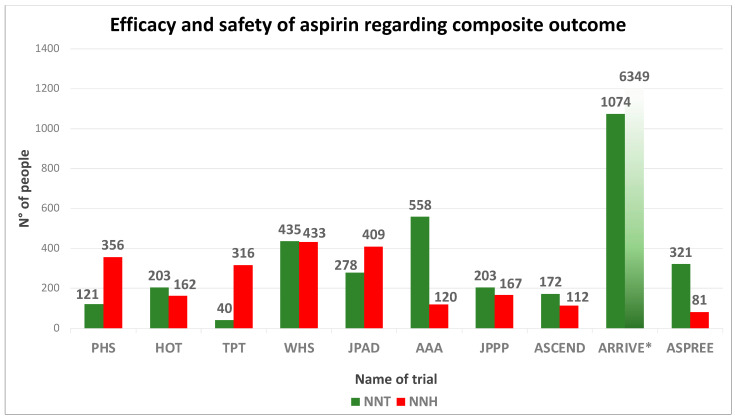
Trade-off between efficacy and safety across trials investigating aspirin use in primary prevention. Numbers needed to treat (NNT) and numbers needed to harm (NNH) displayed in a barplot were calculated as the inverse of absolute risk reduction for a primary efficacy composite endpoint (defined as a composite of fatal and nonfatal myocardial infarction plus fatal and nonfatal ischemic stroke) and major bleeding. * Regarding the “Aspirin to Reduce Risk of Initial Vascular Events” (ARRIVE), aspirin use showed to be protective toward the safety outcome, so this was shown as efficacy data. Data from British Doctor Study (BDS), Primary Prevention Project (PPP), The Prevention Of Progression Of Arterial Disease And Diabetes (POPADAD), and International Polycap Study-3 (TIPS-3) are not reported in this analysis due to lack of data of separate outcome. Abbreviations: PHS: Physicians’ Health Study; HOT: Hypertension Optimal Treatment; TPT: Thrombosis Prevention Trial (exc. Warfarin); WHS: Women’s Health Study; JPAD: Low-dose aspirin for primary prevention of atherosclerotic events in patients with type 2 diabetes; AAA: Aspirin for Asymptomatic Atherosclerosis; JPPP: Japanese Primary Prevention Project; ASCEND: A Study of Cardiovascular Events in Diabetes; ARRIVE: Aspirin to Reduce Risk of Initial Vascular Events ASPREE: Aspirin in Reducing Events in the Elderly.

Historical randomized controlled trials (RCTs) have investigated the use of aspirin in primary prevention, providing initially exciting but not always consistent results (Figure 1 and Figure 2) [14,24,25,26,27,28]. In the Physicians’ Health Study (PHS) [25], 325 mg of aspirin on alternate days decreased the risk of myocardial infarction (MI) by 44% (relative risk—RR—0.56, 95% confidence interval—95% CI—from 0.45 to 0.70, *p* < 0.01) in male physicians aged 40–84 years. The Thrombosis Prevention Trial (TPT) [26], which tested a lower and fixed dose of 75 mg once a day, reported similar findings, as well as the Hypertension Optimal Treatment (HOT) study [14]. However, different results were reported by other trials. 

In the prematurely interrupted Primary Prevention Project (PPP) trial [27], aspirin did not reduce the primary endpoint of cardiovascular death, stroke, or MI (RR 0.90, 95% CI 0.50–1.62, *p* not available) in patients with diabetes mellitus (DM). Aspirin was found not to affect the risk of MI or death from cardiovascular causes (RR 0.91, 95% CI 0.80–1.03, *p* = 0.13) in the Women’s Health Study (WHS) [28], whereas the risk of stroke was reduced (RR 0.83, 95% CI 0.69–0.99, *p* = 0.04).

Subsequently, the Prevention Of Progression of Arterial Disease And Diabetes (POPADAD) trial showed no benefit on cardiovascular events and mortality in adults ≥40 years having type 1 or 2 DM and asymptomatic peripheral arterial disease [29]. The Japanese Primary Prevention of Atherosclerosis With Aspirin for Diabetes (JPAD) trial randomized 2539 Japanese with type-2 DM to low-dose (81 or 100 mg) aspirin or no aspirin [30], showing no reduction in ischemic heart disease, stroke, and peripheral arterial disease (arteriosclerosis obliterans, aortic dissection, mesenteric artery thrombosis) (hazard ratio—HR −1.14, 95% CI 0.91–1.42, *p* = 0.20). 

Pooling these trials, however, a patient-level meta-analysis by the Antithrombotic Trialists’ (ATT) Collaboration showed reduced serious vascular events (non-fatal MI, non-fatal stroke, and cardiovascular death; rate ratio 0.88, 95% CI 0.82–0.94, *p* < 0.01) [31], without differences across pre-specified risk categories. This was mainly driven by a lower risk of non-fatal MI (RR 0.77, 95% CI 0.67–0.89, *p* < 0.01), while no difference was observed in terms of vascular, non-vascular, and all-cause mortality. Of note, however, is the fact that the ischemic benefit was counterbalanced by a significant increase in the risk of major gastrointestinal and other extracranial bleeding (RR 1.54, 95% CI 1.30–1.82, *p* < 0.01), casting doubts on the net advantage of aspirin in unselected patients without significant baseline risk. 

On this basis, subsequent studies focused on populations at higher risk of ischemic events, anticipating a more favourable net clinical benefit. A first attempt at refining patients’ selection in a “modern” cohort was conducted in the Aspirin to Reduce Risk of Initial Vascular Events (ARRIVE) trial [32]. The investigators selected a cohort of 12,546 patients aged ≥55 years (men) or ≥60 years (women) at moderate cardiovascular risk (defined as 10–20% 10-year risk of coronary artery disease, CAD) and with no bleeding risk features. The trial still showed no reduction in the composite of MI or unstable angina, cardiovascular death, stroke, or transient ischemic attack (HR 0.96, 95% CI 0.81–1.13, *p* = 0.60) in the low-dose aspirin arm. However, in this study, there was not an increase in haemorrhagic stroke risk, despite an increase in mild gastric bleedings (HR 2.11, 95% CI 1.36–3.28, *p* = 0.95). A different high-risk subset was selected in the placebo-controlled ASPirin in Reducing Events in the Elderly (ASPREE) trial [33], enrolling 19,114 healthy community-dwelling seniors (≥70 years-old) from Australia and the United States of America. Analysing a composite outcome of fatal CAD, nonfatal MI, fatal or nonfatal stroke, or hospitalization for heart failure, the study found no differences between the two arms (HR 0.95, 95% CI 0.83–1.08, *p* not available), without any differences in each subgroup analysis that may influence cardiovascular risk, but an increase in major bleeding was observed (HR 1.38, 95% CI 1.18–1.62, *p* not available). This trial also revealed a higher risk of all-cause mortality in the aspirin arm (HR 1.14, 95% CI 1.01–1.29, *p* = 0.03) with cancer-related death being the principal aetiology. Finally, in the A Study of Cardiovascular Events in Diabetes (ASCEND) trial [34], the authors evaluated the use of 100 mg of aspirin in 15,000 adults over 40 years old with any type of DM. The composite of MI, stroke or transient ischemic attack, or death from any vascular cause was less frequent in aspirin users (RR 0.88, 95% CI 0.79–0.97, *p* = 0.01), but again counterbalanced by a higher risk of major haemorrhagic events (RR 1.29, 95% CI 1.09–1.52, *p* < 0.01).

Although subsequent meta-analyses continued to show a benefit of aspirin on ischemic events [35], the one from Shah et al. also highlighted a time-dependent heterogeneity in RCT results [36]. In this study, while the benefit of aspirin was preserved in RCTs dating back to the late 1980s and 1990s, it appeared attenuated in more recent studies, suggesting that the lack of modern preventive approaches (e.g., a widespread use of lipid-lowering drugs) could have magnified the efficacy found in earlier studies. Another meta-analysis also supported the lack of ischemic benefit in the elderly, a worrisome finding given the significant proneness to major bleeding of this vulnerable subgroup [37].

The most recent meta-analysis published in 2022 by the U.S. Preventive Services Task Force (USPSTF; N = 134,470) [38], also including data from the long-term follow-up of the JPAD trial and the WHS trial [39,40], confirmed a reduction in the composite of nonfatal MI, nonfatal stroke, and cardiovascular mortality (odds ratio—OR—0.90, 95% CI 0.85–0.95, I square—I^2^—0%); however, no differences were observed in cardiovascular and all-cause mortality up to 10 years. Bleeding risk excess (OR 1.44, 95% CI, 1.32–1.57, I^2^  =  4.7%) was consistently reported across several safety endpoints, including extracranial haemorrhage, major gastrointestinal bleeding, and most importantly intracranial haemorrhage. Of interest, time-to-event analysis revealed an accrual of bleeding by the first year and more pronounced ischemic benefit between 1 and 2 years.

## 4. Looking for a Scapegoat: Can Subgroup Effects Account for the Lack of Benefit?

A variety of settings have been associated with an exaggerated pro-thrombotic milieu and resistance to aspirin. These have been recurrently called into question to address the lack of ischemic benefit, and, as a result, an even less favourable safety–efficacy balance in RCTs. Subgroups characterized by aspirin resistance include diabetics, the obese, women, post-MI, and post-coronary artery bypass graft patients [41]. Since the last two subgroups do not fulfil the criteria for primary prevention, we discuss the first three.

### 4.1. Diabetics

Patient with diabetes who have a two- to four-fold higher risk of ischemic events compared to non-diabetic patients, have been frequently suggested as the most suitable candidates for primary cardiovascular prevention [42]. However, the results of trials have been conflicting. Neither the JPAD nor the ASCEND trials provided convincing support for the use of aspirin in this subgroup [30,34]. In the latter study [34], aspirin met the primary efficacy endpoint, but the benefit was offset by the occurrence of major bleeding. In the meta-analysis by Zheng et al. [35], data for the diabetic population were reported in 10 RCTs (N = 30,448), and the authors only found a borderline significant trend towards less cardiovascular mortality, nonfatal MI, and nonfatal stroke with aspirin (HR 0.90, 95% CI 0.82–1.00, I^2^ = 0%). On the other hand, major bleedings (HR 1.29, 95% CI 1.11–1.51, I^2^ = 0%), particularly major gastrointestinal haemorrhages (HR 1.35, 95% CI 1.05–1.75, I^2^ = 1%), were increased in the experimental arm, whereas intracranial bleedings were not (HR 1.21, 95% CI 0.84–1.76, I^2^ = 1%). The disappointing results may be due to the pathophysiological specificities of diabetic patients. These patients have platelets with unique biological features, including a more rapid platelet turnover and enhanced platelet reactivity, which makes them more prone to thrombotic events [43].

Among the peculiarities of these patients, studies have also shown early recovery of thromboxane synthesis, implying that inhibition of thromboxane-dependent platelet aggregation is not fully maintained for 24 h [44]. On this basis, a potential benefit of reducing aspirin dosing intervals has been suggested. This was indeed supported by several studies, which pointed out greater platelet inhibition when low-dose aspirin was administered twice a day, as opposed to an increased dose administered once a day [43,44,45,46,47,48,49,50]. Unfortunately, this pharmacological advantage is not easily translated into a clear clinical benefit; many other biological mechanisms are involved, including hyperglycaemia itself which contributes to reduced platelet sensitivity [51]. Overall, RCTs on this subgroup of patients are lacking to support a convincing benefit. As a result, diabetic patients are not currently candidates for “ad hoc” schemes.

### 4.2. Obese

Obesity is associated with platelet hyper-reactivity, inflammation, oxidative stress, and endothelial dysfunction [52]. It has also been demonstrated that a higher body mass mitigates some of the platelet inhibition properties of aspirin. A seminal study from Cox et al. showed a direct correlation between body weight and the likelihood of treatment failure [53], as well as inappropriate thromboxane inhibition, whereas others have advocated for improved responsiveness after bariatric surgery and weight loss [54]. While the administration of higher doses of aspirin has been discouraged due to poor additive benefit and safety concerns, reducing intervals between dose administration has been proposed as a more effective approach. Indeed, Petrucci et al. reported a reduction in response to once-daily low-dose aspirin and in vivo platelet activation in obese people [55]. Despite these intriguing pathophysiological data, the improved efficacy of non-conventional aspirin regimens for CVD prevention (e.g., doubled daily administration) in individuals with obesity has not been unequivocally proven, and current guidelines do not recommend adjusting the scheme based on body weight.

### 4.3. Women

Platelets in women exhibit the same or even a greater decrease in reactivity while on aspirin therapy, although the residual platelet reactivity is modestly higher than that observed in men [56,57].

The potential differences in cardiovascular outcomes driven by this heterogeneity have been subject of investigation in previous studies. A subgroup analysis of the ATT Collaboration meta-analysis [31], which incorporated data from the HOT, PPP, and WHS trials [14,27,28], did not reveal significant interactions between sex and treatment effects. This was also the case in subgroup analyses from the ARRIVE, ASPREE and ASCEND trials [32,33,34]. However, real-world data from a recent retrospective, propensity score-matched study indicated a blunted aspirin benefit in women with respect to total cardiovascular events (HR 0.66, 95% CI 0.63–0.69, *p* not available) [58]. In a subanalysis of the WHS trial [28], the occurrence of major cardiovascular events (death from cardiovascular causes, nonfatal MI, or stroke) was significantly reduced only among women aged 65 years or older (RR 0.74, 95% CI 0.59–0.92, *p* = 0.01), primarily driven by a reduction in MI (RR 0.66, 95% CI 0.44–0.97, *p* = 0.04). This finding was confirmed by the extended study follow-up, which highlighted a benefit of low-dose aspirin in this subgroup at 15 years [40]. Therefore, current data supporting a differential clinical effect of aspirin according to sex are limited.

## 5. Aspirin in Primary Prevention and Cancer

Antithrombotics have been called into question to explain non-cardiovascular impact in several clinical settings [59,60]. Initially supported by the observation of tumor growth suppression by NSAIDs in experimental animal models [61], the hypothesis of a potential therapeutic role for aspirin in patients with cancer was then corroborated by observational data [62,63], paving the way for further investigations, with special attention to the field of colorectal cancer (CRC). 

The initial report of two large-scale RCT mentioned above, the WHS and the PHS [28,64], showed no benefit of aspirin on cancer risk in the context of primary prevention, and the Colorectal Adenoma/Carcinoma Prevention Programme 2 (CAPP2) reported similar findings in a selected cohort of patients with Lynch syndrome (resulting in enhanced predisposition to CRC) over a mean follow-up of 2.5 years [65]. However, these observations were later confuted by the extended observational follow-up of the WHS as well as the long-term results of the CAPP2, which consistently showed lower risk of CRC after >10 years in both populations (HR 0.80, 95% CI 0.67–0.97, *p* = 0.02 and HR 0.65, 95% CI 0.43–0.97, *p* = 0.03, respectively) [66,67]. Subsequently, several studies have investigated this topic, but the high heterogeneity regarding study design, the dosage of aspirin, assumption schemes (e.g., daily or every other day), and follow-up duration have led to conflicting results.

A patient-level meta-analysis including RCT of low-dose aspirin in primary and secondary CVD prevention (N = 14,033) suggested lower risk of CRC (HR 0.62, 95% CI 0.43–0.94, *p* < 0.01) and mortality (HR 0.48, 95% CI 0.30–0.77, *p* < 0.01) over a mean follow-up of 18.3 years and after a median duration of treatment of 6 years [68]. According to the same analysis, aspirin was especially beneficial for cancers localized in the proximal colon, which are, moreover, less detectable by endoscopic exploration. Another patient-level pooled analysis (N = 17,285) proved aspirin to also be beneficial in preventing metastases, resulting in lower risk of fatal adenocarcinoma, adenocarcinoma with metastasis at initial diagnosis, and new metastasis diagnosis in patients with localised neoplasia [69]. Interestingly, this benefit was equally reported across different dose regimens, suggesting low-dose aspirin to be effective enough for the purpose of chemoprevention [69]. The value of aspirin for long-term chemoprevention was also confirmed by a large study-level meta-analysis pooling 88 cohort studies and seven RCT, which again confirmed a latency period of at least 10 years with a scheduled treatment duration of at least 5 years [70]. On the heels of these observations, the 2016 USPSTF recommendation statement emphasized the role of aspirin also in CRC prevention, alongside cardiovascular risk reduction, and suggested to start low-dose aspirin treatment in individuals aged 50 to 59 years at high cardiovascular risk able to take the medication daily for at least 10 years, leveraging on the necessity for a long-lasting drug exposure [71]. These recommendations were later questioned following the publication of the ASPREE trial [33], in which aspirin was associated with higher risk of overall mortality in elderly patients, driven primarily by cancer-related deaths. A subsequent analysis did not show an increase in overall cancer incidence; however, higher incidence of cancers at stage IV at diagnosis (HR 1.22, 95% CI 1.02–1.45, *p* not available) and higher mortality in advanced cancer (in stages III: HR 2.11, 95% CI 1.03–4.33, *p* not available; and in stage IV: HR 1.31, 95% CI 1.04–1.64, *p* not available) were observed [72]. 

Although these results need to be further contextualized in the current extensive cancer screening programs (specifically extensive colonoscopy screening programs), they contributed to the downgrade of the class of recommendation in favour of aspirin, also as chemoprotective, by USPSTF in 2022 [38]. Of note, considering the more encouraging findings of the CAPP-2 study, the 2020 National Institute of Health and Care Excellence (NICE) guidelines suggest instead the consideration of aspirin assumption for at least 2 years in patients with Lynch syndrome [73].

There is also evidence that the heterogeneous biology of cancer cells and several common pathophysiological mechanisms underlying cardiovascular disease play a role in determining the effectiveness of aspirin [74]. In a large analysis pooling pathological and clinical data from the Nurses’ Health Study (NHS) and the Health Professionals Follow-up Study (HPFS) [75,76,77], aspirin reduced the risk of developing CRC with significant overexpression of COX-2, a molecular pattern associated with the increase in cancer progression and worse prognosis [78,79]. 

Finally, whilst most observations mentioned so far pertain to CRC prevention/progression, some authors have speculated that other cancers might be equally positively affected by aspirin use, including oesophageal and breast [80]. To our knowledge, this hypothesis remains presently speculative and hardly transferrable to clinical practice.

## 6. Guideline Recommendations

Current recommendations on aspirin use in primary prevention are summarised in Table 1. 

The American College of Cardiology (ACC) and the American Heart Association (AHA) have put forth guidelines in 2019 that suggest considering low-dose aspirin (75–100 mg orally daily) in primary prevention [81]. This recommendation is specifically for selected adults aged between 40 and 70 years who are at high risk of ischemic events, provided they do not meet the criteria for high bleeding risk. On the other hand, the USPSTF advises against the use of aspirin in primary prevention for adults aged 60 years and above [82]. However, they propose a tailored approach for those aged between 40 and 59 years with an estimated 10-year risk of 10% or more. The European Society of Cardiology (ESC) guidelines suggest that aspirin may be considered for primary prevention in patients with DM at high or very high cardiovascular risk, provided there are no clear contraindications [83]. However, they advise against the use of aspirin in individuals at low-to-moderate ischemic risk. The American Diabetes Association (ADA) guidelines propose that aspirin (75–162 mg/day) may be considered as a primary prevention strategy in patients with type 2 diabetes mellitus who are at increased cardiovascular risk [84]. In regard to peripheral artery disease, the latest ESC guidelines endorse low-dose aspirin use in asymptomatic carotid artery stenosis of 50% or more at low bleeding risk [85]. However, they contraindicate aspirin in asymptomatic lower-extremity artery disease.

In summary, aspirin should be considered in subgroups at high risk of ischemic events and low risk of bleeding. Accurate cardiovascular risk stratification is crucial for determining aspirin use. The method for evaluating cardiovascular risk remains an important consideration.

## 7. Considerations for a Modern Approach to Cardiovascular Primary Prevention

This primary prevention is dynamic, and the strength of therapeutic intervention is influenced by how cardiovascular risk is assessed, stratified, and modified by evolving therapies and lifestyles. The focus is not just on platelet activation and thrombotic predisposition, but also on leveraging a wide range of pathophysiological pathways and therapeutic targets through various therapeutic approaches. These approaches include intensive lipid-lowering therapies and tight glycaemic control, among others. The interplay between evolving stratification tools and the availability of increasingly powerful and cost-effective therapies targeting alternative pathways is of particular interest. Notably, an adequate stratification is warranted both for ischemic and haemorrhagic events. The appropriateness of aspirin administration in the setting of primary prevention is a topic that continues to garner attention. This is due to the need to balance the potential benefits of aspirin in reducing ischemic events against the potential risks, such as bleeding. As such, it is crucial to continue researching and discussing these topics to optimize primary prevention strategies.

### 7.1. Defining the Threshold of Intervention

Cardiovascular risk stratification is a complex process that currently relies on three main factors: (i) anatomical and/or functional evidence of atherosclerotic disease, (ii) the presence of specific clinical conditions such as DM, and (iii) the estimated long-term risk of cardiovascular events based on risk scores. While the first two factors are relatively straightforward, score-based risk assessment is inherently flawed due to the time-varying predictive accuracy of risk determinants and the limited generalizability of the score models to specific patient subgroups. 

In the ARRIVE and ASCEND trials [32,34], patients with a higher predicted cardiovascular risk showed a lower risk reduction with aspirin, arguably due to the highly prevalent adoption of other preventive approaches such as aggressive cholesterol reduction. There might also be a progressive attenuation of traditional factor-related cardiovascular risk in the elderly [86,87]. This has resulted in the 2021 ESC guidelines endorsing two different risk stratification tools [83], SCORE2 and SCORE2-OP [88,89], the latter dedicated to people aged over 70 years, to which SCORE2 diabetes was recently added specifically for diabetic patients [90] and a specific add-on tool was developed to improve risk prediction in patients with CKD [91]. There is also geographical heterogeneity across the perceived degree of risk. For instance, while the European guidelines stratify the cardiovascular risk also by age [83] the US counterpart includes in the highest risk stratum only patients with estimated 10-year rates of events greater than 20% regardless of age [81]. These inconsistencies can be reconciled by understanding that the same risk factors may lead to different levels of risk and could result in different class of recommendation among different guidelines. When applied to the specific instance of aspirin administration in primary prevention, this means that regardless of the stratification tool used, the applicability to the individual patient should be ascertained and the safety–efficacy balance should be evaluated. In an editorial of European Heart Journal, Tokgozoglu raised concerns about moving from the average risk level of study subgroups to applicability on the individual [92]. Moreover, she proposed to integrate the present models with other currently disregarded risk factors, genomics and proteomics data, and imaging and biomarker data, hinting at a potential role for artificial intelligence and wearable devices. 

Consideration of non-traditional factors not routinely accounted for by risk stratification tools is also gaining importance. These include not only clinical factors such as BMI, family history, menopause, and comorbidities such as kidney disease, but also environmental, social, and behavioural variables such as stress, physical activity level, and exposure to pollution (Table 2). These are increasingly recognized as risk modifiers in cardiovascular disease [93]. Novel risk markers, such as kidney function, metabolic impairment, and social indexes, have been included in the PREVENT score recently proposed by AHA to refine risk assessment in primary prevention [94]. 

Lipoprotein(a) [Lp(a)] is an established risk factor for atherosclerosis not included in the most used risk scores which presents some peculiarities, including the non-availability, until recently, of specific drugs for its reduction [95,96]. In individuals without CVD but with Lp(a) > 50 mg/dL, aspirin use has been found to be associated with a 46% reduction (HR 0.54, 95% CI 0.32–0.94, *p* = 0.03) in CAD risk in the Multi-Ethnic Study of Atherosclerosis (MESA) prospective cohort study [97]. This result is strengthened with findings from the The Third National Health and Nutrition Examination Survey (NHANES III), in which, after multivariable analysis, regular aspirin use was found to be associated with a 52% lower risk of CAD mortality among those with elevated Lp(a) (HR = 0.48, 95% CI 0.28–0.83, *p* = 0.01), but not for those without elevated Lp(a) (HR = 1.01, 95% CI 0.81–1.25, *p* < 0.01) [98]. Bleedings were not explored in the latter study; however, the bleeding rate was higher among aspirin users in the MESA study (17.5% vs 12.5%, *p* < 0.01), although there was no association with Lp(a) levels after multivariable adjustment. Therefore, further research is needed to investigate the net clinical benefit in this clinical condition.

In addition, other morpho-functional parameters have shown promising results. Examples include the quantification of coronary artery calcium (CAC) and the assessment of platelet reactivity. The former is a marker of subclinical atherosclerosis and can be quantified using the CAC score from routine non-contrast-enhanced coronary computerized tomography [99]. High CAC values independently predict an increased risk of cardiovascular events and mortality [100,101,102], and observational studies have suggested a net clinical benefit of aspirin in subjects aged under 70 years with CAC greater than or equal to 100 [103,104,105]. 

Exaggerated platelet reactivity has been investigated as a potential therapeutic target in specific subgroups such as obese patients. However, there is significant confusion with respect to the diagnosis, definition, and consequently the clinical prevalence of this phenomenon [106]. Functional and genetic testing is increasingly being used for tailored antithrombotic medicine [107,108], and the extension of this approach to primary prevention might potentially play a role in selected patients at risk of cardiovascular events. 

**Figure 3:**
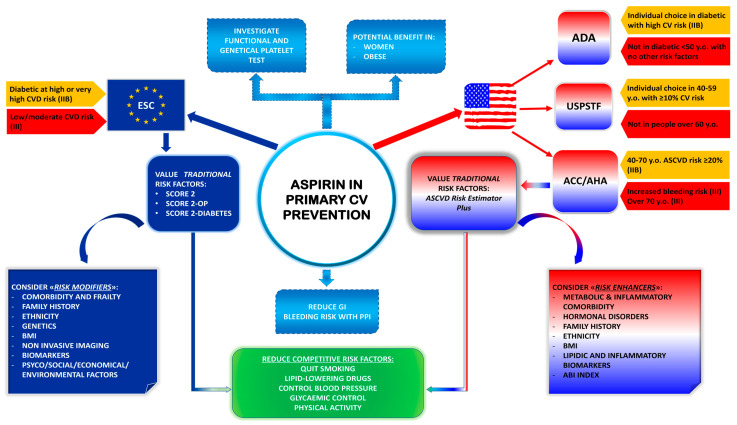
Aspirin in primary cardiovascular prevention: current evidence and guidelines recommendations. Abbreviations: ABI: ankle-brachial index; ACC/AHA: American College of Cardiology/American Heart Association; ADA: American Diabetes Association; ASCVD: atherosclerotic cardiovascular disease; BMI: body mass index; CV: cardiovascular; CVD: cardiovascular disease ESC: European Society of Cardiology; PPI: proton pump inhibitors; USPSTF: United States Preventive Services Task Force; y.o.: years old.

Non-traditional risk contributors, such as the aforementioned, are nowadays recognized as “risk enhancers”. From a practical perspective, they cannot independently support risk stratification. However, they are helpful when the individual’s cardiovascular risk is close to a decision threshold, as indicated in Figure 3.

For example, a study defined the presence of three or more risk enhancers as an optimum threshold for incremental prediction, as it identifies intermediate-risk patients that will benefit from statin therapy [109]. The generalizability of this approach to aspirin administration in primary prevention, however, has not been conclusively proved yet.

### 7.2. Competing Benefit of Alternative Therapies

The widespread adoption of increasingly potent drugs for cardiovascular prevention that target non-thrombotic pathways is a critical factor in reinterpreting the use of aspirin in the context of contemporary primary prevention. Meta-analyses have demonstrated that aspirin is more effective in reducing cardiovascular events in patients who are not adequately treated with hypolipidemic drugs [37]. It is reasonable and biologically plausible to extend this observation to other pathogenetic targets such as hypertension and glycaemic control.

The interplay between lipid-lowering/blood pressure-lowering medication and aspirin was examined in The International Polycap Study 3 (TIPS-3) trial [110]. Over an average follow-up of 4.6 years, the combination of a polypill (containing 40 mg of simvastatin, 100 mg of atenolol, 25 mg of hydrochlorothiazide, and 10 mg of ramipril) and aspirin, compared to placebo, reduced the composite of death from cardiovascular causes, MI, stroke, resuscitated cardiac arrest, heart failure, or revascularization by 31% (HR 0.69, 95% CI 0.50–0.97, *p* not available) in patients without prior events and at intermediate cardiovascular risk. A trend towards a benefit remained, but was not statistically significant, when the polypill without aspirin was compared to placebo (HR 0.79, 95% CI 0.63–1.00, *p* not available). This suggests that the addition of aspirin might be key for risk reduction even in the setting of strong and multi-directional prevention.

However, a study-level meta-analysis pooling 16 trials showed an exceeding risk of major bleeding compared to ischemic benefit across cardiovascular risk strata and regardless of statin administration. This implies that the net benefit of routinely combining statins and aspirin remains questionable [111].

### 7.3. Potential “Facilitators” of Aspirin-Based Primary Prevention

The safety–efficacy profile of aspirin can be enhanced not only by selecting cohorts with a higher ischemic risk but also by minimizing the predisposition to bleeding. Given that most aspirin-related bleeding occurs in the gastrointestinal tract, potential strategies include the eradication of Helicobacter Pylori and the use of proton pump inhibitors (PPIs) [112].

In the COMPASS trial [113], PPI, compared to placebo, reduced gastroduodenal bleeding by 48% in patients taking a combination of rivaroxaban and aspirin (HR 0.52, 95% CI 0.28–0.94, *p* = 0.03). However, the number needed to treat was high (NNT = 982, 95% CI 609–2528).

Specific data on the use of aspirin as monotherapy in primary prevention are currently lacking. Therefore, the large-scale implementation of these strategies would require careful consideration of cost-effectiveness. At present, guidelines recommend PPIs in patients receiving antiplatelet medication only when there is a high risk of gastrointestinal bleeding [83].

## 8. Conclusions

The role of aspirin in targeting thrombosis has been a cornerstone of primary cardiovascular prevention for many years and continues to be a customary practice in clinical settings. However, current data do not universally support aspirin use in primary prevention for the general population. By contrast, they suggest that certain individuals with specific characteristic may still benefit from it. The careful prediction of the expected net benefit is crucial. This requires not only the evaluation of individual risk through traditional risk factors included in available risk stratification tools, but also considering non-traditional risk enhancers and risk modifiers that can aid in the reclassification of patients.

Despite intriguing pathophysiological data, there is currently no conclusive evidence to support a clinically significant interaction by subgroups such as sex or diabetes mellitus. Therefore, further research is needed to fully understand the implications and potential benefits of aspirin use in these specific subgroups.

## Figures and Tables

**Table 1:** Current guideline recommendation. Abbreviation: ASCVD: Atherosclerotic cardiovascular disease; CVD: cardiovascular disease; DM: diabetes mellitus; LEAD: lower-extremity artery disease; N.A.: not available; SAPT: single antiplatelet therapy.

Guidelines	Recommendation	Class	Level
2019 American College of Cardiology/American Heart Association (ACC/AHA) Guideline on the Primary Prevention of Cardiovascular Disease	Low-dose aspirin (75–100 mg orally daily) might be considered for the primary prevention of ASCVD among select adults 40 to 70 years of age who are at higher ASCVD risk but not at increased bleeding risk.	IIb	A
	Low-dose aspirin (75–100 mg orally daily) should not be administered on a routine basis for the primary prevention of ASCVD among adults > 70 years of age.	III	B-R
	Low-dose aspirin (75–100 mg orally daily) should not be administered for the primary prevention of ASCVD among adults of any age who are at increased risk of bleeding.	III	C-LD
2023 American Diabetes Association (ADA) “Standards of Medical Care in Diabetes”	Aspirin therapy (75–162 mg/day) may be considered as a primary prevention strategy in those with diabetes who are at increased cardiovascular risk, after a comprehensive discussion with the patient on the benefits versus the comparable increased risk of bleeding.	IIb	A
	Aspirin is not recommended for those at low risk of ASCVD (such as men and women, aged < 50 years old with diabetes with no other major ASCVD risk factors) as the low benefit is likely to be outweighed by the risks of bleeding.	III	N.A.
2022 Aspirin Use to Prevent Cardiovascular Disease: US Preventive Services Task Force (USPSTF) Recommendation Statement	The decision to initiate low-dose aspirin use for the primary prevention of CVD in adults aged 40 to 59 years old who have a 10% or greater 10-year CVD risk should be an individual one. Evidence indicates that the net benefit of aspirin use in this group is small. Persons who are not at increased risk for bleeding and are willing to take low-dose aspirin daily are more likely to benefit.	N.A.	C
	The USPSTF recommends against initiating low-dose aspirin use for the primary prevention of CVD in adults 60 years or older.	III	D
2021 European Society of Cardiology (ESC) Guidelines on cardiovascular disease prevention in clinical practice	In patients with DM at high or very high CVD risk, low-dose aspirin may be considered for primary prevention in the absence of clear contraindications.	IIb	A
	Antiplatelet therapy is not recommended in individuals with low/moderate CVD risk due to the increased risk of major bleeding.	III	A
2017 ESC Guidelines on the Diagnosis and Treatment of Peripheral Arterial Diseases, in collaboration with the European Society for Vascular Surgery (ESVS)	In patients with symptomatic carotid stenosis, long-term SAPT is recommended.	I	A
	In patients with asymptomatic > 50% carotid artery stenosis, long-term antiplatelet therapy (commonly low-dose aspirin) should be considered when the bleeding risk is low.	IIa	C
	Long-term SAPT is recommended in symptomatic patients with LEAD.	I	A
	Because of a lack of proven benefit, antiplatelet therapy is not routinely indicated in patients with isolated asymptomatic LEAD.	III	A

**Table 2:** Traditional cardiovascular risk factors and novel risk enhancers or risk modifiers according to latest ACC/AHA and ESC guidelines. Abbreviations: ABI: ankle-brachial index; ACC/AHA: American College of Cardiology/American Heart Association; apoB: apolipoprotein B; AF: atrial fibrillation; ASCVD: atherosclerotic cardiovascular disease; CAC: coronary artery calcium score; CCTA: contrast computed tomography coronary angiography; CKD: chronic kidney disease; COPD: chronic obstructive pulmonary disease; HIV/AIDS: human immudeficiency virus/ acquired immune deficiency syndrome; HF: heart failure IMT: intima-media thickness; Lp(a): lipoprotein (a).

Traditional Cardiovascular Risk Factors	
Age	Sex
Blood pressure	Blood cholesterol
Cigarette smoking	Diabetes Mellitus
Adiposity	Lifestyle (nutrition and physical activity)
**2019 ACC/AHA *risk enhancers***	**2021 ESC *risk modifiers***
Family history of premature ASCVD	Family history
Primary hypercholesterolemia	Genetics
Metabolic syndrome	Body composition
Chronic kidney disease	Frailty
History of premature menopause and history of pregnancy-associated conditions that increase later ASCVD risk	Imaging (CAC, CCTA, IMT, ABI)
High-risk race/ethnicity	Ethnicity
Chronic inflammatory conditions (psoriasis, lupus, RA, HIV/AIDS, etc.)	Biomarkers
Lipids/biomarkers: associated with increased ASCVD risk	Psychosocial factors
Persistently elevated, primary hypertriglyceridemia (≥175 mg/dL)	Socioeconomic determinants
Elevated high-sensitivity C-reactive protein (≥2.0 mg/L)	Environmental exposure
Elevated Lp(a): ≥50 mg/dL or ≥125 nmol/L constitutes a risk-enhancing factor especially at higher levels of Lp(a)	Clinical conditions (CKD, AF, COPD, Cancer, HF, inflammatory conditions, infections, sleeping and mental disorders)
Elevated apoB: ≥130 mg/dL corresponds to an LDL-C ≥ 160 mg/dL and constitutes a risk-enhancing factor	
ABI < 0.9	

## References

[1] Edney P., Jackson P., Burrell B., Lawton N., Leigh N., Lindsay McLellan P., Newman C., Pickard J., Weiser R., Williams S. (1988). Secondary Prevention of Vascular Disease by Prolonged Antiplatelet Treatment. Br. Med. J. (Clin. Res. Ed.).

[2] Benenati S., Galli M., De Marzo V., Pescetelli F., Toma M., Andreotti F., Della Bona R., Canepa M., Ameri P., Crea F. (2021). Very Short vs. Long Dual Antiplatelet Therapy after Second Generation Drug-Eluting Stents in 35 785 Patients Undergoing Percutaneous Coronary Interventions: A Meta-Analysis of Randomized Controlled Trials. Eur. Heart J. Cardiovasc. Pharmacother..

[3] Benenati S., Crimi G., Canale C., Pescetelli F., De Marzo V., Vergallo R., Galli M., Della Bona R., Canepa M., Ameri P. (2022). Duration of Dual Antiplatelet Therapy and Subsequent Monotherapy Type in Patients Undergoing Drug-Eluting Stent Implantation: A Network Meta-Analysis. Eur. Heart J. Cardiovasc. Pharmacother..

[4] O’Brien C.W., Juraschek S.P., Wee C.C. (2019). Prevalence of Aspirin Use for Primary Prevention of Cardiovascular Disease in the United States: Results from the 2017 National Health Interview Survey. Ann. Intern. Med..

[5] Capodanno D., Angiolillo D.J. (2016). Aspirin for Primary Cardiovascular Risk Prevention and Beyond in Diabetes Mellitus. Circulation.

[6] Patrono C., García Rodríguez L.A., Landolfi R., Baigent C. (2005). Low-Dose Aspirin for the Prevention of Atherothrombosis. N. Engl. J. Med..

[7] FitzGerald G.A. (1991). Mechanisms of Platelet Activation: Thromboxane A2 as an Amplifying Signal for Other Agonists. Am. J. Cardiol..

[8] Moncada S., Vane J.R. (1979). Arachidonic Acid Metabolites and the Interactions between Platelets and Blood-Vessel Walls. N. Engl. J. Med..

[9] Kobayashi T., Tahara Y., Matsumoto M., Iguchi M., Sano H., Murayama T., Arai H., Oida H., Yurugi-Kobayashi T., Yamashita J.K. (2004). Roles of Thromboxane A(2) and Prostacyclin in the Development of Atherosclerosis in ApoE-Deficient Mice. J. Clin. Investig..

[10] Patrignani P., Filabozzi P., Patrono C. (1982). Selective Cumulative Inhibition of Platelet Thromboxane Production by Low-Dose Aspirin in Healthy Subjects. J. Clin. Investig..

[11] Mcadam B.F., Catella-Lawson F., Mardini I.A., Kapoor S., Lawson J.A., Fitzgerald G.A. (1999). Systemic Biosynthesis of Prostacyclin by Cyclooxygenase (COX)-2: The Human Pharmacology of a Selective Inhibitor of COX-2. Proc. Natl. Acad. Sci. USA.

[12] FitzGerald G.A., Oates J.A., Hawiger J., Maas R.L., Roberts L.J., Lawson J.A., Brash A.R. (1983). Endogenous Biosynthesis of Prostacyclin and Thromboxane and Platelet Function during Chronic Administration of Aspirin in Man. J. Clin. Investig..

[13] Patrono C., Ciabarroni G., Patrignani P., Pugliese F., Filabozzi P., Catella F., Davi G., Forni L. (1985). Clinical Pharmacology of Platelet Cyclooxygenase Inhibition. Circulation.

[14] Hansson L., Zanchetti A., Carruthers S.G., Dahlöf B., Elmfeldt D., Julius S., Ménard J., Rahn K.H., Wedel H., Westerling S. (1998). Effects of Intensive Blood-Pressure Lowering and Low-Dose Aspirin in Patients with Hypertension: Principal Results of the Hypertension Optimal Treatment (HOT) Randomised Trial. Lancet.

[15] Snowden S., Nelson R. (2011). The Effects of Nonsteroidal Anti-Inflammatory Drugs on Blood Pressure in Hypertensive Patients. Cardiol. Rev..

[16] Reilly I., FitzGerald G. (1987). Inhibition of Thromboxane Formation In Vivo and Ex Vivo: Implications for Therapy With Platelet Inhibitory Drugs. Blood.

[17] Maree A.O., Curtin R.J., Dooley M., Conroy R.M., Crean P., Cox D., Fitzgerald D.J. (2005). Platelet Response to Low-Dose Enteric-Coated Aspirin in Patients With Stable Cardiovascular Disease. J. Am. Coll. Cardiol..

[18] Denis M.M., Tolley N.D., Bunting M., Schwertz H., Jiang H., Lindemann S., Yost C.C., Rubner F.J., Albertine K.H., Swoboda K.J. (2005). Escaping the Nuclear Confines: Signal-Dependent Pre-MRNA Splicing in Anucleate Platelets. Cell.

[19] Evangelista V., Manarini S., Di Santo A., Capone M.L., Ricciotti E., Di Francesco L., Tacconelli S., Sacchetti A., D’Angelo S., Scilimati A. (2006). De Novo Synthesis of Cyclooxygenase-1 Counteracts the Suppression of Platelet Thromboxane Biosynthesis by Aspirin. Circ. Res..

[20] Cagnina A., Chabot O., Davin L., Lempereur M., Maréchal P., Oury C., Lancellotti P. (1999). Atherosclerosis—An Inflammatory Disease. N. Engl. J. Med..

[21] Lindemann S., Tolley N.D., Dixon D.A., McIntyre T.M., Prescott S.M., Zimmerman G.A., Weyrich A.S. (2001). Activated Platelets Mediate Inflammatory Signaling by Regulated Interleukin 1β Synthesis. J. Cell Biol..

[22] Brown J.R., DuBois R.N. (2005). COX-2: A Molecular Target for Colorectal Cancer Prevention. J. Clin. Oncol..

[23] Thun M.J., Jacobs E.J., Patrono C. (2012). The Role of Aspirin in Cancer Prevention. Nat. Rev. Clin. Oncol..

[24] Peto R., Gray R., Collins R., Wheatley K., Hennekens C., Jamrozik K., Warlow C., Hafner B., Thompson E., Norton S. (1988). Randomised Trial of Prophylactic Daily Aspirin in British Male Doctors. Br. Med. J. (Clin. Res. Ed.).

[25] Steering Committee of the Physicians’ Health Study Research Group (1989). Final Report on the Aspirin Component of the Ongoing Physicians’ Health Study. N. Engl. J. Med..

[26] Meade T.W., Wilkes H.C., Kelleher C.C., Roderick P.J., Brennan P.J., Wilson C.W., Howarth D.J., Stirling Y., Garrow K., Dickinson C.J. (1998). Thrombosis Prevention Trial: Randomised Trial of Low-Intensity Oral Anticoagulation with Warfarin and Low-Dose Aspirin in the Primary Prevention of Ischaemic Heart Disease in Men at Increased Risk. Lancet.

[27] Sacco M., Pellegrini F., Roncaglioni M.C., Avanzini F., Tognoni G., Nicolucci A. (2003). Primary Prevention of Cardiovascular Events with Low-Dose Aspirin and Vitamin E in Type 2 Diabetic Patients: Results of the Primary Prevention Project (PPP) Trial. Diabetes Care.

[28] Ridker P.M., Cook N.R., Lee I.-M., Gordon D., Gaziano J.M., Manson J.E., Hennekens C.H., Buring J.E. (2005). A Randomized Trial of Low-Dose Aspirin in the Primary Prevention of Cardiovascular Disease in Women. N. Engl. J. Med..

[29] Belch J., MacCuish A., Campbell I., Cobbe S., Taylor R., Prescott R., Lee R., Bancroft J., MacEwan S., Shepherd J. (2008). The Prevention of Progression of Arterial Disease and Diabetes (POPADAD) Trial: Factorial Randomised Placebo Controlled Trial of Aspirin and Antioxidants in Patients with Diabetes and Asymptomatic Peripheral Arterial Disease. Br. Med. J..

[30] Ogawa H., Nakayama M., Morimoto T., Uemura S., Kanauchi M., Doi N., Jinnouchi H., Sugiyama S., Saito Y. (2008). Low-Dose Aspirin for Primary Prevention of Atherosclerotic Events in Patients with Type 2 Diabetes: A Randomized Controlled Trial. J. Am. Med. Assoc..

[31] Collins R., Peto R., Hennekens C., Doll R., Bubes V., Buring J., Dushkesas R., Gaziano M., Brennan P., Meade T. (2009). Aspirin in the Primary and Secondary Prevention of Vascular Disease: Collaborative Meta-Analysis of Individual Participant Data from Randomised Trials. Lancet.

[32] Gaziano J.M., Brotons C., Coppolecchia R., Cricelli C., Darius H., Gorelick P.B., Howard G., Pearson T.A., Rothwell P.M., Ruilope L.M. (2018). Use of Aspirin to Reduce Risk of Initial Vascular Events in Patients at Moderate Risk of Cardiovascular Disease (ARRIVE): A Randomised, Double-Blind, Placebo-Controlled Trial. Lancet.

[33] McNeil J.J., Wolfe R., Woods R.L., Tonkin A.M., Donnan G.A., Nelson M.R., Reid C.M., Lockery J.E., Kirpach B., Storey E. (2018). Effect of Aspirin on Cardiovascular Events and Bleeding in the Healthy Elderly. N. Engl. J. Med..

[34] Bowman L., Mafham M., Wallendszus K., Stevens W., the ASCEND Study Collaborative Group Effects of Aspirin for Primary Prevention in Persons with Diabetes Mellitus (2018). Effects of Aspirin for Primary Prevention in Persons with Diabetes Mellitus. N. Engl. J. Med..

[35] Zheng S.L., Roddick A.J. (2019). Association of Aspirin Use for Primary Prevention with Cardiovascular Events and Bleeding Events: A Systematic Review and Meta-Analysis. J. Am. Med. Assoc..

[36] Shah R., Khan B., Latham S.B., Khan S.A., Rao S.V. (2019). A Meta-Analysis of Aspirin for the Primary Prevention of Cardiovascular Diseases in the Context of Contemporary Preventive Strategies. Am. J. Med..

[37] Galli M., Andreotti F., D’amario D., Vergallo R., Montone R.A., Porto I., Crea F. (2020). Aspirin in Primary Prevention of Cardiovascular Disease in the Elderly. Eur. Heart J. Cardiovasc. Pharmacother..

[38] Guirguis-Blake J.M., Evans C.V., Perdue L.A., Bean S.I., Senger C.A. (2022). Aspirin Use to Prevent Cardiovascular Disease and Colorectal Cancer: Updated Evidence Report and Systematic Review for the US Preventive Services Task Force. J. Am. Med. Assoc..

[39] Saito Y., Okada S., Ogawa H., Soejima H., Sakuma M., Nakayama M., Doi N., Jinnouchi H., Waki M., Masuda I. (2017). Low-Dose Aspirin for Primary Prevention of Cardiovascular Events in Patients with Type 2 Diabetes Mellitus: 10-Year Follow-Up of a Randomized Controlled Trial. Circulation.

[40] Cook N.R., Cole S.R., Buring J.E. (2012). Aspirin in the Primary Prevention of Cardiovascular Disease in the Women’s Health Study: Effect of Noncompliance. Eur. J. Epidemiol..

[41] Ardeshna D., Khare S., Jagadish P.S., Bhattad V., Cave B., Khouzam R.N. (2019). The Dilemma of Aspirin Resistance in Obese Patients. Ann. Transl. Med..

[42] Dal Canto E., Ceriello A., Rydén L., Ferrini M., Hansen T.B., Schnell O., Standl E., Beulens J.W.J. (2019). Diabetes as a Cardiovascular Risk Factor: An Overview of Global Trends of Macro and Micro Vascular Complications. Eur. J. Prev. Cardiol..

[43] Capodanno D., Patel A., Dharmashankar K., Ferreiro J.L., Ueno M., Kodali M., Tomasello S.D., Capranzano P., Seecheran N., Darlington A. (2011). Pharmacodynamic Effects of Different Aspirin Dosing Regimens in Type 2 Diabetes Mellitus Patients with Coronary Artery Disease. Circ. Cardiovasc. Interv..

[44] Spectre G., Arnetz L., Östenson C.G., Brismar K., Li N., Hjemdahl P. (2011). Twice Daily Dosing of Aspirin Improves Platelet Inhibition in Whole Blood in Patients with Type 2 Diabetes Mellitus and Micro-or Macrovascular Complications. Thromb. Haemost..

[45] DiChiara J., Bliden K.P., Tantry U.S., Hamed M.S., Antonino M.J., Suarez T.A., Bailon O., Singla A., Gurbel P.A. (2007). The Effect of Aspirin Dosing on Platelet Function in Diabetic and Nondiabetic Patients: An Analysis from the Aspirin-Induced Platelet Effect (ASPECT) Study. Diabetes.

[46] Gurbel P.A., Bliden K.P., DiChiara J., Newcomer J., Weng W., Neerchal N.K., Gesheff T., Chaganti S.K., Etherington A., Tantry U.S. (2007). Evaluation of Dose-Related Effects of Aspirin on Platelet Function: Results from the Aspirin-Induced Platelet Effect (ASPECT) Study. Circulation.

[47] Addad F., Chakroun T., Elalamy I., Abderazek F., Chouchene S., Dridi Z., Gerotziafas G.T., Hatmi M., Hassine M., Gamra H. (2010). Antiplatelet Effect of Once- or Twice-Daily Aspirin Dosage in Stable Coronary Artery Disease Patients with Diabetes. Int. J. Hematol..

[48] Dillinger J.G., Drissa A., Sideris G., Bal Dit Sollier C., Voicu S., Manzo Silberman S., Logeart D., Drouet L., Henry P. (2012). Biological Efficacy of Twice Daily Aspirin in Type 2 Diabetic Patients with Coronary Artery Disease. Am. Heart J..

[49] Bethel M.A., Harrison P., Sourij H., Sun Y., Tucker L., Kennedy I., White S., Hill L., Oulhaj A., Coleman R.L. (2016). Randomized Controlled Trial Comparing Impact on Platelet Reactivity of Twice-Daily with Once-Daily Aspirin in People with Type 2 Diabetes. Diabet. Med..

[50] Jones W.S., Mulder H., Wruck L.M., Pencina M.J., Kripalani S., Muñoz D., Crenshaw D.L., Effron M.B., Re R.N., Gupta K. (2021). Comparative Effectiveness of Aspirin Dosing in Cardiovascular Disease. N. Engl. J. Med..

[51] Pulcinelli F.M., Biasucci L.M., Riondino S., Giubilato S., Leo A., Di Renzo L., Trifir E., Mattiello T., Pitocco D., Liuzzo G. (2009). COX-1 Sensitivity and Thromboxane A2 Production in Type 1 and Type 2 Diabetic Patients under Chronic Aspirin Treatment. Eur. Heart J..

[52] Santilli F., Vazzana N., Liani R., Guagnano M.T., Davì G. (2012). Platelet Activation in Obesity and Metabolic Syndrome. Obes. Rev..

[53] Cox D., Maree A.O., Dooley M., Conroy R., Byrne M.F., Fitzgerald D.J. (2006). Effect of Enteric Coating on Antiplatelet Activity of Low-Dose Aspirin in Healthy Volunteers. Stroke.

[54] Norgard N.B., Monte S.V., Fernandez S.F., Ma Q. (2017). Aspirin Responsiveness Changes in Obese Patients Following Bariatric Surgery. Cardiovasc. Ther..

[55] Petrucci G., Zaccardi F., Giaretta A., Cavalca V., Capristo E., Cardillo C., Pitocco D., Porro B., Schinzari F., Toffolo G. (2019). Obesity Is Associated with Impaired Responsiveness to Once-Daily Low-Dose Aspirin and in Vivo Platelet Activation. J. Thromb. Haemost..

[56] Becker D.M., Segal J., Vaidya D., Yanek L.R., Herrera-Galeano J.E., Bray P.F., Moy T.F., Becker L.C., Faraday N. (2006). Sex Differences in Platelet Reactivity and Response to Low-Dose Aspirin Therapy. J. Am. Med. Assoc..

[57] Qayyum R., Becker D.M., Yanek L.R., Moy T.F., Becker L.C., Faraday N., Vaidya D. (2008). Platelet Inhibition by Aspirin 81 and 325 Mg/Day in Men versus Women without Clinically Apparent Cardiovascular Disease. Am. J. Cardiol..

[58] Kim S.P., Ryu J., Kim S.H., Yoon H.J. (2023). Low-Dose Aspirin in the Primary Prevention of Cardiovascular Diseases: A Retrospective, Propensity Score Matched Study. Atherosclerosis.

[59] Cattaneo M., Faioni E.M. (2012). Why Does Ticagrelor Induce Dyspnea?. Thromb. Haemost..

[60] Benenati S., Canale C., De Marzo V., Della Bona R., Rosa G.M., Porto I. (2021). Atrial Fibrillation and Alzheimer’s Disease: A Conundrum. Eur. J. Clin. Investig..

[61] Pollard M., Luckert P.H. (1981). Effect of Indomethacin on Intestinal Tumors Induced in Rats by the Acetate Derivative of Dimethylnitrosamine. Science.

[62] Thun M.J., Namboodiri M.M., Heath C.W. (1991). Aspirin Use and Reduced Risk of Fatal Colon Cancer. N. Engl. J. Med..

[63] Kune G., Kune S., Watson L.F. (1988). Colorectal Cancer Risk, Chronic Illnesses, Operations, and Medications: Case Control Results from the Melbourne Colorectal Cancer Study. Cancer Res..

[64] Gann P.H., Manson J.E., Glynn R.J., Buring J.E., Hennekens C.H. (1993). Low-Dose Aspirin and Incidence of Colorectal Tumors in a Randomized Trial. J. Natl. Cancer Inst..

[65] Burn J., Bishop D.T., Mecklin J.-P., Macrae F., Möslein G., Olschwang S., Bisgaard M.-L., Ramesar R., Eccles D., Maher E.R. (2008). Effect of Aspirin or Resistant Starch on Colorectal Neoplasia in the Lynch Syndrome. N. Engl. J. Med..

[66] Cook N.R., Lee I.M., Zhang S.M., Moorthy M.V., Buring J.E. (2013). Alternate-Day, Low-Dose Aspirin and Cancer Risk: Long-Term Observational Follow-up of a Randomized Trial. Ann. Intern. Med..

[67] Burn J., Gerdes A.M., MacRae F., Mecklin J.P., Moeslein G., Olschwang S., Eccles D., Evans D.G., Maher E.R., Bertario L. (2011). Long-Term Effect of Aspirin on Cancer Risk in Carriers of Hereditary Colorectal Cancer: An Analysis from the CAPP2 Randomised Controlled Trial. Lancet.

[68] Rothwell P.M., Wilson M., Elwin C.E., Norrving B., Algra A., Warlow C.P., Meade T.W. (2010). Long-Term Effect of Aspirin on Colorectal Cancer Incidence and Mortality: 20-Year Follow-up of Five Randomised Trials. Lancet.

[69] Rothwell P.M., Wilson M., Price J.F., Belch J.F.F., Meade T.W., Mehta Z. (2012). Effect of Daily Aspirin on Risk of Cancer Metastasis: A Study of Incident Cancers during Randomised Controlled Trials. Lancet.

[70] Wang L., Zhang R., Yu L., Xiao J., Zhou X., Li X., Song P., Li X. (2021). Aspirin Use and Common Cancer Risk: A Meta-Analysis of Cohort Studies and Randomized Controlled Trials. Front. Oncol..

[71] Bibbins-Domingo K., Grossman D.C., Curry S.J., Davidson K.W., Epling J.W., García F.A.R., Gillman M., Harper D.M., Kemper A.R., Krist A.H. (2016). Aspirin Use for the Primary Prevention of Cardiovascular Disease and Colorectal Cancer: U.S. Preventive Services Task Force Recommendation Statement. Ann. Intern. Med..

[72] Mcneil J.J., Gibbs P., Orchard S.G., Lockery J.E., Bernstein W.B., Cao Y., Ford L., Haydon A., Kirpach B., Macrae F. (2021). Effect of Aspirin on Cancer Incidence and Mortality in Older Adults. J. Natl. Cancer Inst..

[73] National Institute for Health and Care (2020). Effectiveness of Aspirin in the Prevention of Colorectal Cancer in People with Lynch Syndrome.

[74] Di Fusco S.A., Cianfrocca C., Bisceglia I., Spinelli A., Alonzo A., Mocini E., Gulizia M.M., Gabrielli D., Oliva F., Imperoli G. (2022). Potential Pathophysiologic Mechanisms Underlying the Inherent Risk of Cancer in Patients with Atherosclerotic Cardiovascular Disease. Int. J. Cardiol..

[75] Giovannucci E., Egan K.M., Hunter D.J., Stampfer M.J., Colditz G.A., Willett W.C., Speizer F.E. (1995). Aspirin and the Risk of Colorectal Cancer in Women. N. Engl. J. Med..

[76] Giovannucci E., Rimm E.B., Stampfer M.J., Colditz G.A., Ascherio A., Willett W.C. (1994). Aspirin Use and the Risk for Colorectal Cancer and Adenoma in Male Health Professionals. Ann. Intern. Med..

[77] Chan A.T., Ogino S., Fuchs C.S. (2007). Aspirin and the Risk of Colorectal Cancer in Relation to the Expression of COX-2. N. Engl. J. Med..

[78] Shechan K.M., Shcahan K., O’Donoghue P., Imacswecney F., Conroy R.M., Fitzgerald D.J., Murray F.E. (1999). The Relationship Between Cyclooxygenase-2 Expression and Colorectal Cancer. J. Am. Med. Assoc..

[79] Ogino S., Kirkner G.J., Nosho K., Irahara N., Kure S., Shima K., Hazra A., Chan A.T., Dehari R., Giovannucci E.L. (2008). Cyclooxygenase-2 Expression Is an Independent Predictor of Poor Prognosis in Colon Cancer. Clin. Cancer Res..

[80] González-Pérez A., García Rodríguez L.A., López-Ridaura R. (2003). Effects of Non-Steroidal Anti-Inflammatory Drugs on Cancer Sites Other than the Colon and Rectum: A Metal-Analysis. BMC Cancer.

[81] Arnett D.K., Blumenthal R.S., Albert M.A., Buroker A.B., Goldberger Z.D., Hahn E.J., Himmelfarb C.D., Khera A., Lloyd-Jones D., McEvoy J.W. (2019). 2019 ACC/AHA Guideline on the Primary Prevention of Cardiovascular Disease: A Report of the American College of Cardiology/American Heart Association Task Force on Clinical Practice Guidelines. Circulation.

[82] Davidson K.W., Barry M.J., Mangione C.M., Cabana M., Chelmow D., Coker T.R., Davis E.M., Donahue K.E., Jaén C.R., Krist A.H. (2022). Aspirin Use to Prevent Cardiovascular Disease: US Preventive Services Task Force Recommendation Statement. J. Am. Med. Assoc..

[83] Visseren F.L.J., MacH F., Smulders Y.M., Carballo D., Koskinas K.C., Bäck M., Benetos A., Biffi A., Boavida J.M., Capodanno D. (2021). 2021 ESC Guidelines on Cardiovascular Disease Prevention in Clinical PracticeDeveloped by the Task Force for Cardiovascular Disease Prevention in Clinical Practice with Representatives of the European Society of Cardiology and 12 Medical Societies with the Special Contribution of the European Association of Preventive Cardiology (EAPC). Eur. Heart J..

[84] American Diabetes Association Professional Practice Committee (2022). 10. Cardiovascular Disease and Risk Management: Standards of Medical Care in Diabetes—2022. Diabetes Care.

[85] Aboyans V., Ricco J.B., Bartelink M.L.E.L., Björck M., Brodmann M., Cohnert T., Collet J.P., Czerny M., De Carlo M., Debus S. (2018). 2017 ESC Guidelines on the Diagnosis and Treatment of Peripheral Arterial Diseases, in Collaboration with the European Society for Vascular Surgery (ESVS) Document Covering Atherosclerotic Disease of Extracranial Carotid and Vertebral, Mesenteric, Renal, Upper and Lower Extremity ArteriesEndorsed by: The European Stroke Organization (ESO)The Task Force for the Diagnosis and Treatment of Peripheral Arterial Diseases of the European Society of Cardiology (ESC) and of the European Society for Vascul. Eur. Heart J..

[86] Ahmadi S.F., Streja E., Zahmatkesh G., Streja D., Kashyap M., Moradi H., Molnar M.Z., Reddy U., Amin A.N., Kovesdy C.P. (2015). Reverse Epidemiology of Traditional Cardiovascular Risk Factors in the Geriatric Population. J. Am. Med. Dir. Assoc..

[87] Vaes B., Depoortere D., Van Pottelbergh G., Matheï C., Neto J., Degryse J. (2017). Association between Traditional Cardiovascular Risk Factors and Mortality in the Oldest Old: Untangling the Role of Frailty. BMC Geriatr..

[88] Hageman S., Pennells L., Ojeda F., Kaptoge S., Kuulasmaa K., de Vries T., Xu Z., Kee F., Chung R., SCORE2 working group and ESC Cardiovascular risk collaboration (2021). SCORE2 Risk Prediction Algorithms: New Models to Estimate 10-Year Risk of Cardiovascular Disease in Europe. Eur. Heart J..

[89] de Vries T.I., Cooney M.T., Selmer R.M., Hageman S.H.J., Pennells L.A., Wood A., Kaptoge S., Xu Z., Westerink J., SCORE2-OP working group and ESC Cardiovascular risk collaboration (2021). SCORE2-OP Risk Prediction Algorithms: Estimating Incident Cardiovascular Event Risk in Older Persons in Four Geographical Risk Regions. Eur. Heart J..

[90] Pennells L., Kaptoge S., Østergaard H.B., Read S.H., Carinci F., Franch-Nadal J., Petitjean C., Taylor O., Hageman S.H.J., Xu Z. (2023). SCORE2-Diabetes: 10-Year Cardiovascular Risk Estimation in Type 2 Diabetes in Europe. Eur. Heart J..

[91] Matsushita K., Kaptoge S., Hageman S.H.J., Sang Y., Ballew S.H., Grams M.E., Surapaneni A., Sun L., Arnlov J., Bozic M. (2023). Including Measures of Chronic Kidney Disease to Improve Cardiovascular Risk Prediction by SCORE2 and SCORE2-OP. Eur. J. Prev. Cardiol..

[92] Tokgozoglu L., Torp-Pedersen C. (2021). Redefining Cardiovascular Risk Prediction: Is the Crystal Ball Clearer Now?. Eur. Heart J..

[93] Montone R.A., Camilli M., Calvieri C., Magnani G., Bonanni A., Bhatt D.L., Rajagopalan S., Crea F., Niccoli G. (2024). Exposome in Ischaemic Heart Disease: Beyond Traditional Risk Factors. Eur. Heart J..

[94] Khan S.S., Coresh J., Pencina M.J., Ndumele C.E., Rangaswami J., Chow S.L., Palaniappan L.P., Sperling L.S., Virani S.S., Ho J.E. (2023). Novel Prediction Equations for Absolute Risk Assessment of Total Cardiovascular Disease Incorporating Cardiovascular-Kidney-Metabolic Health: A Scientific Statement From the American Heart Association. Circulation.

[95] Di Fusco S.A., Arca M., Scicchitano P., Alonzo A., Perone F., Gulizia M.M., Gabrielli D., Oliva F., Imperoli G., Colivicchi F. (2022). Lipoprotein(a): A Risk Factor for Atherosclerosis and an Emerging Therapeutic Target. Heart.

[96] Reyes-Soffer G., Ginsberg H.N., Berglund L., Duell P.B., Heffron S.P., Kamstrup P.R., Lloyd-Jones D.M., Marcovina S.M., Yeang C., Koschinsky M.L. (2022). Lipoprotein(a): A Genetically Determined, Causal, and Prevalent Risk Factor for Atherosclerotic Cardiovascular Disease: A Scientific Statement From the American Heart Association. Arterioscler. Thromb. Vasc. Biol..

[97] Bhatia H.S., Trainor P., Carlisle S., Tsai M.Y., Criqui M.H., Defilippis A., Tsimikas S. (2024). Aspirin and Cardiovascular Risk in Individuals with Elevated Lipoprotein(a): The Multi-Ethnic Study of Atherosclerosis. J. Am. Heart Assoc..

[98] Razavi A.C., Richardson L.C., Coronado F., Dzaye O., Bhatia H.S., Mehta A., Quyyumi A.A., Vaccarino V., Budoff M.J., Nasir K. (2024). Aspirin Use for Primary Prevention Among US Adults with and without Elevated Lipoprotein(a). Am. J. Prev. Cardiol..

[99] Agatston A.S., Janowitz W.R., Hildner F.J., Zusmer N.R., Viamonte M., Detrano R. (1990). Quantification of Coronary Artery Calcium Using Ultrafast Computed Tomography. J. Am. Coll. Cardiol..

[100] Arad Y., Spadaro L.A., Goodman K., Newstein D., Guerci A.D. (2000). Prediction of Coronary Events with Electron Beam Computed Tomography. J. Am. Coll. Cardiol..

[101] Detrano R., Guerci A.D., Carr J.J., Bild D.E., Burke G., Folsom A.R., Liu K., Shea S., Szklo M., Bluemke D.A. (2008). Coronary Calcium as a Predictor of Coronary Events in Four Racial or Ethnic Groups. N. Engl. J. Med..

[102] Razavi A.C., Shaw L.J., Berman D.S., Budoff M.J., Wong N.D., Vaccarino V., van Assen M., De Cecco C.N., Quyyumi A.A., Mehta A. (2023). Left Main Coronary Artery Calcium and Diabetes Confer Very-High-Risk Equivalence in Coronary Artery Calcium >1,000. Cardiovasc. Imaging.

[103] Miedema M.D., Duprez D.A., Misialek J.R., Blaha M.J., Nasir K., Silverman M.G., Blankstein R., Budoff M.J., Greenland P., Folsom A.R. (2014). Use of Coronary Artery Calcium Testing to Guide Aspirin Utilization for Primary Prevention: Estimates from the Multi-Ethnic Study of Atherosclerosis. Circ. Cardiovasc. Qual. Outcomes.

[104] Nasir K., Bittencourt M.S., Blaha M.J., Blankstein R., Agatson A.S., Rivera J.J., Miemdema M.D., Sibley C.T., Shaw L.J., Blumenthal R.S. (2015). Implications of Coronary Artery Calcium Testing Among Statin Candidates According to American College of Cardiology/American Heart Association Cholesterol Management Guidelines: MESA (Multi-Ethnic Study of Atherosclerosis). J. Am. Coll. Cardiol..

[105] Cainzos-Achirica M., Miedema M.D., McEvoy J.W., Al Rifai M., Greenland P., Dardari Z., Budoff M., Blumenthal R.S., Yeboah J., Duprez D.A. (2020). Coronary Artery Calcium for Personalized Allocation of Aspirin in Primary Prevention of Cardiovascular Disease in 2019. Circulation.

[106] Mansour K., Taher A.T., Musallam K.M., Alam S. (2009). Aspirin Resistance. Adv. Hematol..

[107] Galli M., Benenati S., Capodanno D., Franchi F., Rollini F., D’Amario D., Porto I., Angiolillo D.J. (2021). Guided versus Standard Antiplatelet Therapy in Patients Undergoing Percutaneous Coronary Intervention: A Systematic Review and Meta-Analysis. Lancet.

[108] Galli M., Benenati S., Franchi F., Rollini F., Capodanno D., Biondi-Zoccai G., Vescovo G.M., Cavallari L.H., Bikdeli B., Ten Berg J. (2022). Comparative Effects of Guided vs. Potent P2Y12 Inhibitor Therapy in Acute Coronary Syndrome: A Network Meta-Analysis of 61 898 Patients from 15 Randomized Trials. Eur. Heart J..

[109] Akintoye E., Afonso L., Jayanna M.B., Bao W., Briasoulis A., Robinson J. (2021). Prognostic Utility of Risk Enhancers and Coronary Artery Calcium Score Recommended in the 2018 ACC/AHA Multisociety Cholesterol Treatment Guidelines over the Pooled Cohort Equation: Insights from 3 Large Prospective Cohorts. J. Am. Heart Assoc. Cardiovasc. Cerebrovasc. Dis..

[110] Yusuf S., Joseph P., Dans A., Gao P., Teo K., Xavier D., López-Jaramillo P., Yusoff K., Santoso A., Gamra H. (2021). Polypill with or without Aspirin in Persons without Cardiovascular Disease. N. Engl. J. Med..

[111] Safi U., Khan M.M., Ahmad N., Lone M., Neal S., Kleiman M., Adeel Arshad M., Vardhmaan Jain M., Mahmoud Al Rifai M.M., Hassaan B. (2023). Aspirin with or without Statin in Individuals without Atherosclerotic Cardiovascular Disease Across Risk Categories. JACC Adv..

[112] Lanas A., Fuentes J., Benito R., Serrano P., Bajador E., Sáinz R. (2002). Helicobacter Pylori Increases the Risk of Upper Gastrointestinal Bleeding in Patients Taking Low-Dose Aspirin. Aliment. Pharmacol. Ther..

[113] Steffel J., Eikelboom J.W., Anand S.S., Shestakovska O., Yusuf S., Fox K.A.A. (2020). The COMPASS Trial: Net Clinical Benefit of Low-Dose Rivaroxaban plus Aspirin as Compared with Aspirin in Patients with Chronic Vascular Disease. Circulation.

